# Optimization of artificial membrane feeding system for lone star ticks, *Amblyomma americanum* (Acari: Ixodidae), and experimental infection with *Rickettsia amblyommatis* (Rickettsiales: Rickettsiaceae)

**DOI:** 10.1093/jme/tjad158

**Published:** 2023-12-16

**Authors:** Ilia Rochlin, Dennis Chu, Matthew Gmelin, Justin Le, Martha B Furie, David G Thanassi, Hwan Keun Kim

**Affiliations:** Center for Infectious Diseases, Stony Brook University, Stony Brook, NY, USA; Department of Microbiology and Immunology, Stony Brook University, Stony Brook, NY, USA; Center for Infectious Diseases, Stony Brook University, Stony Brook, NY, USA; Department of Microbiology and Immunology, Stony Brook University, Stony Brook, NY, USA; Center for Infectious Diseases, Stony Brook University, Stony Brook, NY, USA; Department of Microbiology and Immunology, Stony Brook University, Stony Brook, NY, USA; Center for Infectious Diseases, Stony Brook University, Stony Brook, NY, USA; Department of Microbiology and Immunology, Stony Brook University, Stony Brook, NY, USA; Center for Infectious Diseases, Stony Brook University, Stony Brook, NY, USA; Department of Pathology, Stony Brook University, Stony Brook, NY, USA; Center for Infectious Diseases, Stony Brook University, Stony Brook, NY, USA; Department of Microbiology and Immunology, Stony Brook University, Stony Brook, NY, USA; Center for Infectious Diseases, Stony Brook University, Stony Brook, NY, USA; Department of Microbiology and Immunology, Stony Brook University, Stony Brook, NY, USA

**Keywords:** artificial feeding, ticks, membrane, *Rickettsia*

## Abstract

With the introduction of siliconized artificial membranes, various artificial feeding systems (AFS) for hard ticks (*Ixodidae*) have been developed over the last decades. Most AFS utilize similar core components but employ diverse approaches, materials, and experimental conditions. Published work describes different combinations of the core components without experimental optimizations for the artificial feeding of different tick species. *Amblyomma americanum* L., (Acari: Ixodidae) (lone star tick) is a known vector and reservoir for diverse tick-borne pathogens, such as *Rickettsia amblyommatis* and *Ehrlichia chaffeensis*. Ongoing environmental changes have supported the expansion of *A. americanum* into new habitats, contributing to increased tick-borne diseases in endemic areas. However, a significant knowledge gap exists in understanding the underlying mechanisms involved in *A. americanum* interactions with tick-borne pathogens. Here, we performed a systematic analysis and developed an optimized AFS for nymphal lone star ticks. Our results demonstrate that Goldbeater’s membranes, rabbit hair, hair extract, and adult lone star ticks significantly improved the attachment rate of nymphal ticks, whereas tick frass and frass extract did not. With the optimized conditions, we achieved an attachment rate of 46 ± 3% and a success rate of 100% (i.e., one or more attached ticks) in each feeding experiment for nymphal lone star ticks. When fed on sheep blood spiked with *R. amblyommatis*, both nymphal and adult lone star ticks acquired and maintained *R. amblyommatis*, demonstrating the feasibility of studying *A. americanum*–pathogen interactions using AFS. Our study can serve as a roadmap to optimize and improve AFS for other medically relevant tick species.

## Introduction

Over the last 3 decades, the development of artificial feeding systems (AFS) for ixodid ticks enabled investigators to study tick biology under various experimental conditions (Waladde et al. 1991, Kröber and Guerin 2007, Crispell et al. 2019, Vimonish et al. 2020, 2021, González et al. 2021, Garcia Guizzo et al. 2023, Sebastian et al. 2023). In particular, the generation of siliconized artificial membranes revolutionized AFS and allowed investigators to use different chemical and biological substances to enhance tick attachment and maintain feeding to repletion (Kröber and Guerin 2007, Andrade et al. 2014). Thus far, different AFS have been successfully developed for approximately 20 tick species (González et al. 2021). The principal components of ixodid AFS include a temperature-regulated feeding chamber, artificial membrane, and chemical and biological attractants/stimulants. Previously developed AFS methods describe significant variability in experimental methods for assembling the feeding chamber, generating artificial membranes, extracting stimulants, and applying additional substances. However, it remains unclear how these changes impact the attachment and feeding rates, impeding efforts to standardize AFS protocols for individual tick species and make these approaches broadly available for investigators in the field. Thus, studies are necessary to determine optimal experimental conditions and achieve consistent attachment and feeding of medically important tick species. The availability of improved AFS will facilitate investigations into the underlying molecular mechanisms involved in the transmission of tick-borne pathogens.

*Rickettsia amblyommatis* are Gram-negative bacteria belonging to the spotted fever group of *Rickettsia* and frequently infect *Amblyomma americanum*, a native tick species with widespread distribution and increasing populations across the eastern United States (Monzón et al. 2016, Sanchez-Vicente et al. 2019, Rochlin et al. 2022). *A. americanum* exhibits aggressive biting behavior and plays an important role in disease ecology as a confirmed vector for rickettsiosis, ehrlichiosis, Southern Tick-Associated Rash Illness, tularemia, and Heartland virus (Paddock and Yabsley 2007). Further, *A. americanum* bites are implicated in red meat allergies, also known as alpha-gal syndrome (Commins et al. 2011, Crispell et al. 2019, Young et al. 2021). Within *A. americanum* ticks, *R. amblyommatis* is found in organ tissues (ovaries, midgut, and salivary glands) suitable for its vertical and horizontal transmission (Zanetti et al. 2008, Levin et al. 2018, Suwanbongkot et al. 2019). Past studies have demonstrated that *R. amblyommatis* causes vascular inflammation and mild disease manifestations in spotted fever animal infection models (Rivas et al. 2015, Snellgrove et al. 2021, Yen et al. 2021). However, the pathogenic potential of *R. amblyommatis* in patients remains to be determined.

While limited information exists, prior studies describe the successful application of AFS for *A. americanum*. For instance, investigators used AFS to characterize the structure and proteomic contents of cement cones formed by *A. americanum* (Bullard et al. 2016). In another study, Li et al. (2019) described the use of an *A. americanum* AFS to characterize the biological roles of inward rectifier potassium channels in regulating salivary gland function and blood feeding. However, these studies did not describe the detailed optimization processes and potential technical difficulties. Here, to address this gap in knowledge, we (i) compared how different attractants influence *A. americanum* attachment rates, (ii) designed optimized AFS for *A. americanum*, (iii) tested whether *A. americanum* nymphs can be infected with *R. amblyommatis* when fed with pathogen-spiked blood using the optimized AFS, and (iv) identified remaining weaknesses and experimental challenges. This study may serve as a roadmap for investigators developing and standardizing AFS for different tick species and facilitate the application of AFS in studying host-pathogen-tick interactions.

## Materials and Methods

### Membrane and Feeding Chamber Assembly

**Fig. 1.**
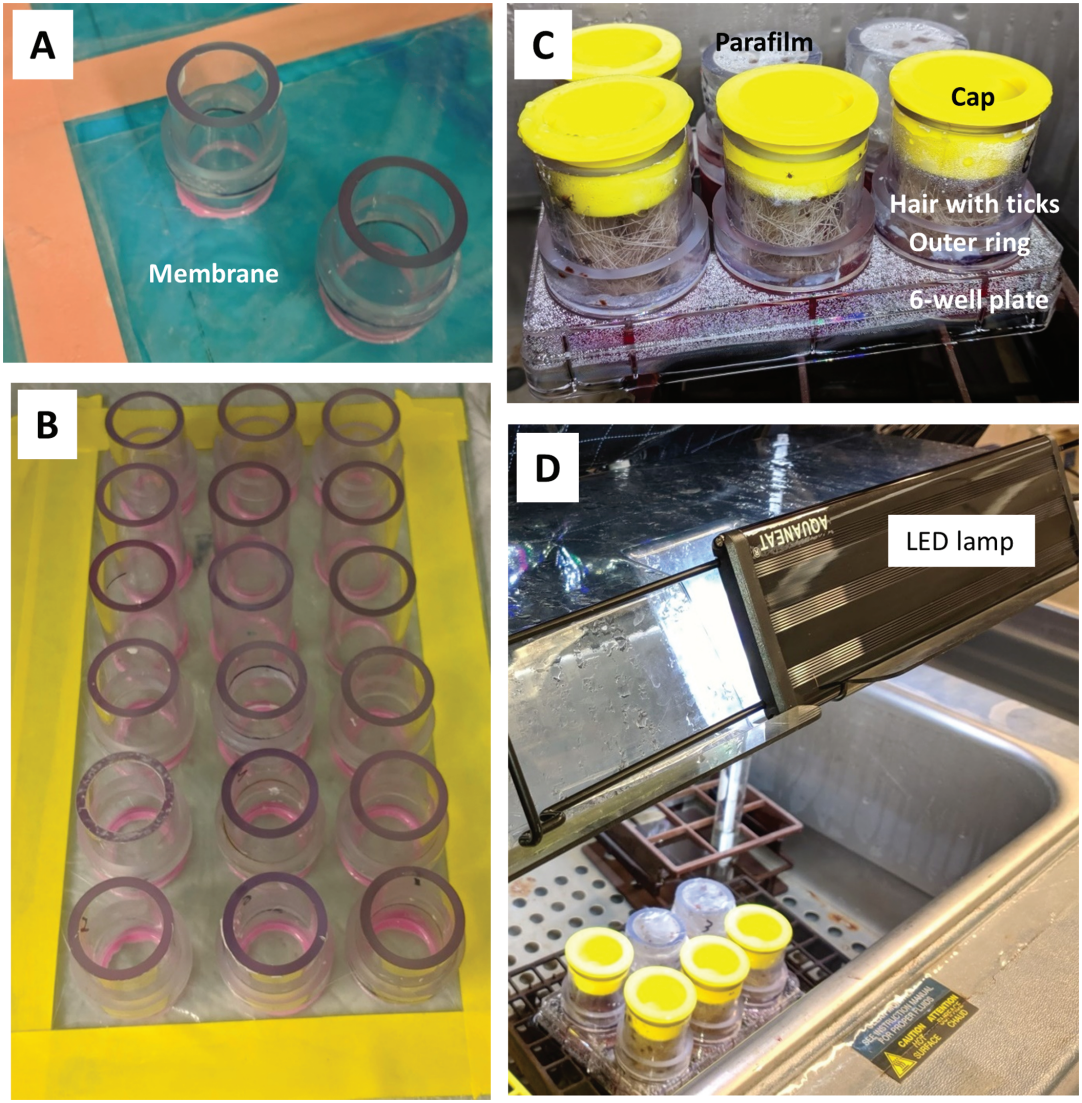
Artificial tick feeding chamber assembly. A) Lens paper membrane with feeding chambers attached using MoldMax 30 silicone glue. The inner chamber and the outer ring are visible. B) Goldbeater’s membrane with attached feeding chambers. The membrane is positioned over plastic wrap on a glass plate. The wrap makes it possible to detach the unit without tearing the membrane off after cutting it out using a scalpel. C) After membrane attachment, the feeding chambers are placed on a 6-well plate. The inner tube holds the host hair and the ticks. The tube is sealed with a plastic cap or parafilm perforated with insect pins to allow air circulation. D) The assembled 6-well plate is placed in a secondary container (not shown for clarity). The assembled unit is placed in a 37°C water bath to maintain temperature and humidity. A photoperiod of 16:8 hours (light:dark) is maintained with an LED lamp connected to a timer on top of the transparent lid. The entire water bath is then covered with a dark cloth.

Silicone membranes were prepared in a fume hood using the following methods (Oliver et al. 2016, Trentelman et al. 2017). Lens paper membranes suitable for adult ticks were made using re-generated cellulose lens paper (6 × 8 inches; Fisher Scientific, Pittsburg, PA) and Smooth-On Ecoflex 00-50 silicone rubber (Smooth-On, Inc., Macungie, PA). Following the manufacturer’s instructions, Ecoflex silicone rubber was mixed with hexane (15% v/v). The single-sided Scotch tape was used to place a sheet of lens cleaning paper on plastic wrap (Fig. 1A). Approximately, 3 ml of the silicone rubber solution was then applied to the lens cleaning paper and spread with a small plastic squeegee to completely cover the paper, removing as much of the remaining silicone as possible with repeated squeezing. For immature tick feeding, Goldbeater’s skin (6 × 22 inches; TALAS, Brooklyn, NY) was used instead of lens cleaning paper, combined with softer Smooth-On Ecoflex 00-10 silicone rubber mixed with hexane (15% v/v). We constructed feeding chambers using clear polycarbonate tubes [inner ring 1/1.25 inches (I.D./O.D.) and outer ring 1.25/1.5 inches (I.D./O.D.); Plastic craft, West Nyack, NY] glued together using clear universal glue (Gorilla).

The silicone-saturated membranes were positioned over plastic wrap on a glass plate and cured for at least 24 h. The wrap made it possible to detach the unit without tearing the membrane off. The feeding chambers were attached to the membrane using either MoldMax 30 silicone glue (Smooth-On, Inc., Macungie, PA) or 100% silicone sealant (Gorilla) by dipping the chamber in the glue or thinly spreading the glue on the chamber’s rim. The chambers were then placed on the membrane, rotated slightly to ensure a good seal, cured for at least 12 h, and cut out using scalpel (Fig. 1A and B). Lens paper membranes were autoclaved before use, whereas Goldbeater’s membranes were surface-sterilized by incubating with 70% ethanol for 15 min in a 6-well plate.

### Membrane Pre-Treatment

Membranes were pre-treated on the “inner” side, that is, where the ticks were subsequently introduced, with different combinations of host hair, host hair extract, tick frass, and tick frass extract (Supplementary Table S1). Hair samples from 2 different mammalian species were used. White-tailed deer (*Odocoileus virginianus*, Zimmerman) hair was obtained by clipping it off a roadkill carcass. Rabbit hair (*Oryctolagus cuniculus*, L.) was obtained from the Division of Laboratory Animal Resources at Stony Brook University.

Two hair extraction methods were tested. The dichloromethane extraction method was employed initially as follows (Krull et al. 2017, Hart et al. 2018, Koci et al. 2018). Raw hair samples were incubated in dichloromethane (1:5 ratio of hair weight to dichloromethane volume) in glass bottles overnight. The solution was distributed into 2 ml tubes and evaporated in a CentriVap Concentrator at 40°C for approximately 2 h. The pellet was resuspended to a final concentration of 7 mg/ml and stored in glass vials at −20°C. Since dichloromethane is reactive with common laboratory plastics, a second extraction method was also tested (Dukes and Rodriguez 1976, Tajeri et al. 2016). Host hair samples were incubated with methanol (1:8 ratio of hair weight to methanol volume) in 50 ml Falcon tubes overnight, then methanol was evaporated to approximately 5% of the original volume using a CentriVap Concentrator at 40°C. The resulting solution was kept in Eppendorf tubes at −20°C. Once extracted, 80 µl solution was applied to the membrane and allowed to evaporate for 4–12 h in a chemical hood.

Tick frass extract was prepared following a previously published protocol (Oliver 2019). Briefly, raw tick frass (Oklahoma State University, tick rearing facility) was dissolved in distilled water (100 mg/ml) containing 1 mM reduced glutathione (GSH), incubated overnight at room temperature, and stored at 4°C. Once extracted, 30 µl solution was spread on a membrane pre-treated with hair extract and allowed to air dry for 2–4 h in a chemical hood.

### Tick Feeding Using AFS

We utilized laboratory-reared (BEI resources, Manassas, VA) and field-collected *A. americanum* ticks (from natural host species such as *Odocoileus virginianus* or the local environment by flagging; Supplementary Table S1). All ticks were maintained at 4°C for up to 3 months. We introduced nymphal ticks to the feeding chamber (5–20 ticks per chamber) containing loosely arranged host hair and a membrane pre-treated with host hair extract and the conspecific frass/extract. For some experiments, we co-housed nymphal ticks with adult ticks to enhance the attachment and feeding of nymphal ticks. Sterile, mechanically defibrinated sheep blood (Hemostat Laboratories, Dixon, CA) was supplemented with 2 ml glucose solution (1 g/ml in sterile water, autoclave-sterilized) per liter of blood to stabilize blood cells (Oliver et al. 2016). At each blood change, the sheep blood was further supplemented with 0.1% 4 mM ATP (Sigma–Aldrich, referred to as complete sheep blood).

The feeding chambers were placed into a 6-well plate, with each well containing 4.5 ml of the complete sheep blood (Fig. 1C). Each feeding chamber was sealed with a cap (WW-12X, Caplugs Inc., Buffalo, NY) or parafilm, which had been perforated using insect pins. The plate was then placed into a secondary plastic container floated inside a water bath heated to 37°C and covered with dark cloth (Fig. 1D). A photoperiod of 16:8 hours (light:dark) was maintained by using an aquarium LED lamp (AQQA aquarium light) connected to a timer (GE 24-h heavy-duty indoor plug-in mechanical timer). Blood in the feeding chambers was replaced every 12–24 h. To replace the blood, the secondary container was surface-sterilized with 70% ethanol and placed in a biosafety cabinet. The feeding chambers were removed from the 6-well plate and rinsed with pre-heated sterile PBS at 37°C. The feeding chambers were then placed in freshly prepared 6-well plates containing pre-warmed complete sheep blood. At the end of experiment, ticks that completed a blood meal and detached were pooled with those that remained attached to the membrane. All ticks were kept in a desiccator with an oversaturated potassium chloride (KCl) solution to maintain approximately 85% relative humidity at room temperature.

### Infection of *A. americanum* Ticks With *R. amblyommatis* Using AFS

Vero cells (African green monkey kidney cells, ATCC) were cultured in Dulbecco’s modified Eagle’s medium (DMEM, Gibco) supplemented with 10% heat-inactivated fetal bovine serum (HI-FBS, Gibco) at 37°C in a 5% CO_2_ atmosphere. Stocks of *R. amblyommatis* strain GAT-30V (Chris Paddock, CDC) were generated by growing rickettsiae in Vero cells at 34°C in a 5% CO_2_ atmosphere. Rickettsiae were purified from Vero cells by disrupting Vero cells with glass beads and a series of centrifugation (1,000 × g to remove Vero cell debris and 17,000 × g to collect rickettsial pellets). The infectious titers were determined by infecting fresh monolayers of Vero cells with 10-fold serial dilutions of purified *R. amblyommatis* in DMEM supplemented with 5% HI-FBS. *A. americanum* ticks were pre-fed on sterile complete sheep blood for 72 h. After pre-feeding, *A. americanum* ticks were exposed to freshly prepared sheep blood containing *R. amblyommatis* (5 × 10^7^ PFU per well) for 60 h, with blood changes occurring every 12 h. Mock infection units received the same sheep blood lacking *R. amblyommatis*. After incubation, unattached and unfed ticks were removed from the feeding chambers, counted, and kept separately. Attached and fed ticks were carefully removed from the membrane and combined with detached engorged ticks, counted, and surface-disinfected by washing with 1% household bleach solution for 1 min, followed by 3 washes with sterile PBS. Then, partially or fully engorged ticks were dried on a paper towel and kept in a desiccator for 5 or 14-days before PCR-testing the presence of *R. amblyommatis*.

### DNA Isolation and PCR-Testing for *R. amblyommatis* Infection

One tick was placed in a 2.0 ml screw cap tube containing approximately 20–25 0.1 mm zirconia beads, 2–3 2.0 mm zirconia beads (Biospec), and 300 µl DNAzol (DN127, Molecular Research Center, Cincinnati, OH). Tick samples were homogenized using a Mini-Beadbeater 16 (Biospec, USA) for 90 s and visually checked for complete homogenization. If needed, the samples were processed again for 90 s (Crowder et al. 2010, Schulze et al. 2013). Tissue fragments were pelleted by centrifugation at 10,000 × g for 10 min at room temperature. For DNA precipitation, 250 μl supernatant was mixed with 125 μl ethanol and centrifuged at 5,000 × g for 10 min. The pellet was washed twice with 200 μl 75% ethanol, resuspended in 50 μl sterile water, and stored at 4°C for PCR analysis.

We used 2 primers (RambFWD: 5ʹ-TTCCTGTAAATAAATGCAAGCCTCT-3ʹ, RambREV: 5ʹ-ATGGCAGTCAACATTACCAAAGC-3ʹ, Integrated DNA Technologies) defining a 157 bp region specific for *R. amblyommatis*. PCR reactions (50 μl) contained 100–1,000 ng template DNA, 2 U of recombinant Z-Taq polymerase (Takara Bio USA, Inc.), 1 × Z-Taq buffer containing 3 mM Mg^2+^, 1 × PCR enhancer (Invitrogen), 0.2 μmol of each primer, and 200 μmol dNTP mix. The following PCR program setting was used to amplify the target region on *R. amblyommatis* chromosomal DNA: 2 min at 98°C, 25 cycles of 5 s at 98°C, 10 s at 53°C, and 5 s at 72°C, followed by 2 min at 72°C. Genomic DNA was prepared from Renografin-purified *R. amblyommatis* GAT-30V and used as a positive control (PureLink Genomic DNA Mini Kit, Invitrogen). After PCR, each sample (10 μl) was analyzed by 1.5% Tris–Acetate EDTA agarose gel electrophoresis with 100 bp DNA ladders (New England Biolabs) and stained with GelRed Nucleic Acid Stain (Sigma).

### Statistical Analysis

Data were analyzed by R v. 4.2.0 (R Development Core Team 2019) to determine statistical significance. Multivariate effects of attachment stimuli, membrane material, and other factors were estimated using a generalized linear model with binomial distribution. The response variable was a binomial variable, with attached ticks representing successes and unattached ticks representing failures. The *P*-values were obtained by likelihood ratio tests comparing the full model with and without the effect in question. The function emmeans (package emmeans) was used for post hoc testing for multiple comparisons using the Tukey method (Lenth 2023). Fitted values, 95% confidence intervals, and comparison arrows (based on the Tukey method) were extracted from the models. Comparison arrows represent Tukey pairwise comparisons between treatments, and where the arrows overlap, there is no significant difference between treatments. Other plots were produced using packages ggplot2 v. 3.3.6.

## Results

### AFS Optimization for *A. americanum* Nymphs

**Fig. 2.**
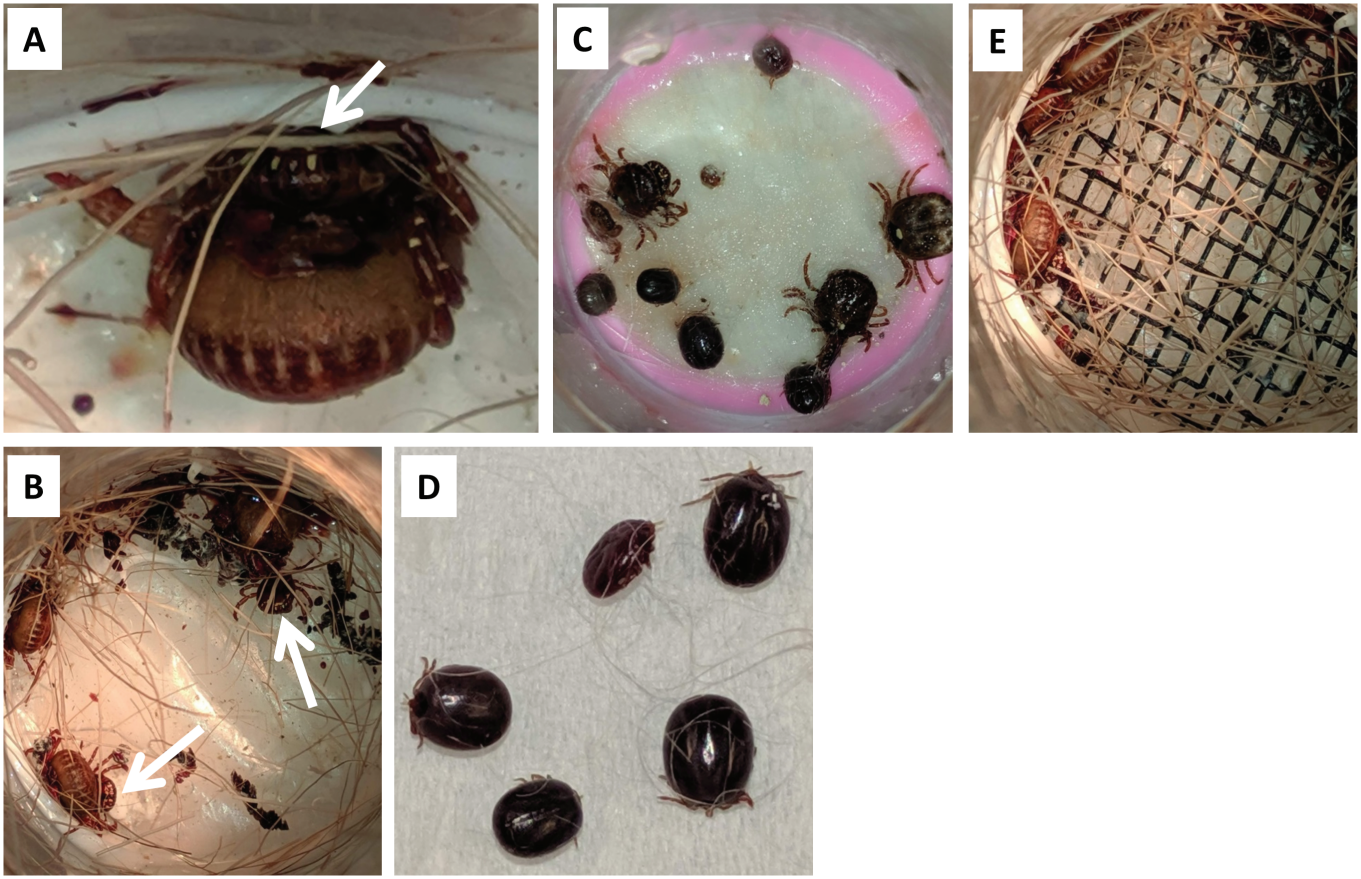
Feeding experiments with *A. americanum*. Host hair was partially or fully removed before photographing the ticks. A and B) Partially engorged *A. americanum* adult ticks attached to lens paper membrane with deer hair/extract. Arrows indicate males attached next to females. Tick frass and remaining host hair are visible. C) *A. americanum* adults and nymphs attached to Goldbeater’s membrane with rabbit hair and extract. Nymphs are attached near feeding adults. D) Fully engorged *A. americanum* nymphs detached from the membrane. E) Partially engorged *A. americanum* adult ticks attached to lens paper membrane with glued fiberglass mosquito mesh and deer hair and extract.

We performed preliminary experiments to determine the attachment rates for 4 different species of nymphs (*A. americanum*, *Ixodes scapularis* Say, *Rhipicephalus sanguineus* Latreille, and *Haemaphysalis longicornis,* Neumann) exposed to different experimental conditions (unpublished data). Of the 4 tick species, *A. americanum* ticks consistently attached to the membranes, with nymphs frequently engorging to repletion (Fig. 2). Thus, we aimed to test experimental conditions for *A. americanum* nymphs by determining the attachment rates of adults and nymphs exposed to different combinations of feeding stimuli and attractants (Table 1).

**Table 1.** AFS experimental conditions tested for *A. americanum* nymphal ticks

With adults^a^	Membrane^b^	Hair^c^	Hair ext^d^	Chemical^e^	Frass^f^	Frass ext^g^	Tick source^h^	Units^i^	Sample size^j^	Mean attachment (%)^k^	SE^l^
N	GB	Rabbit	N	−	N	N	Lab	2	20	40.0	9.5
N	GB	Rabbit	Y	DM	N	N	Lab	2	21	19.1	0.3
N	GB	Rabbit	Y	MT	N	Y	Lab	2	20	30.0	6.3
N	LP	deer	Y	DM	Y	N	Wild	1	20	0.0	−
N	LP	Deer	Y	DM	Y	Y	Wild	1	20	0.0	−
N	LP	Rabbit	Y	DM	Y	Y	Lab	3	30	0.0	0.0
Y	GB	Rabbit	N	−	N	N	Lab	3	31	25.5	4.0
Y	GB	Rabbit	N	−	N	Y	Lab	2	20	35.0	7.9
Y	GB	Rabbit	Y	DM	Y	Y	Lab	2	17	88.9	3.8
Y	GB	Rabbit	Y	MT	N	N	Lab	5	50	46.0	3.3
Y	GB	Rabbit	Y	MT	Y	N	Wild	2	34	29.4	1.4
Y	GB	Rabbit	Y	MT	Y	Y	Lab	6	56	66.7	5.7
Y	LP	Rabbit	Y	DM	N	N	Lab	1	10	80.0	−
Y	LP	Rabbit	Y	MT	N	N	Lab	2	20	15.0	4.7
Y	LP	Rabbit	Y	MT	Y	Y	Lab	3	30	0.0	0.0

^a^Adult ticks were added to the nymphal units (Y: yes and N: no).

^b^Membrane materials used for experiments: Goldbeater’s skin (GB) or lens paper (LP).

^c^Hair samples obtained from 2 host species: rabbit and deer.

^d^Application of hair extract solution to the membrane.

^e^Chemicals used for extraction: dichloromethane (DM) or methanol (MT).

^f^Application of tick frass to the feeding chamber.

^g^Application of tick frass extract solution to the membrane.

^h^Source of ticks: laboratory-reared (lab) or field-caught (wild).

^i^Total number of AFS units.

^j^Total number of ticks.

^k^Tick attachment rate in percent.

^l^Standard error of the estimate.

#### Membrane materials.

**Fig. 3.**
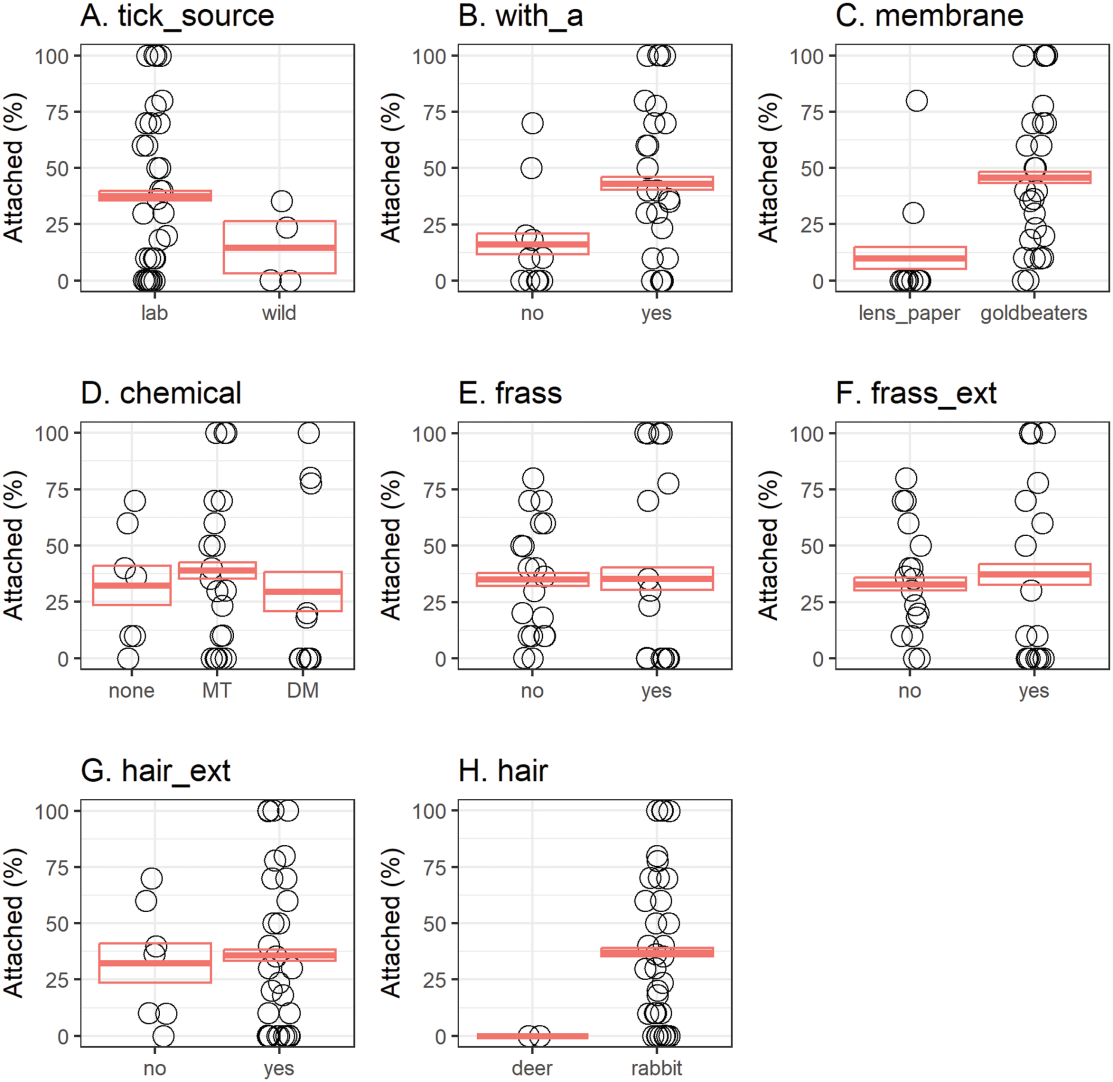
Bivariate analysis comparing *A. americanum* nymph attachment rates of the units assembled with A) different sources of *A. americanum* ticks [lab (laboratory-reared) and wild (field-collected)], B) adult *A. americanum* ticks, C) Goldbeater’s skin or lens paper membrane material, D) hair extract solutions prepared by different chemicals [DM (dichloromethane) and MT (methanol)], E) application of tick frass, F) application of tick frass extract, G) application of hair extract, and H) deer or rabbit hair. Open circles indicate individual AFS units. The crossbar shows the mean and 95% confidence intervals.

**Fig. 4.**
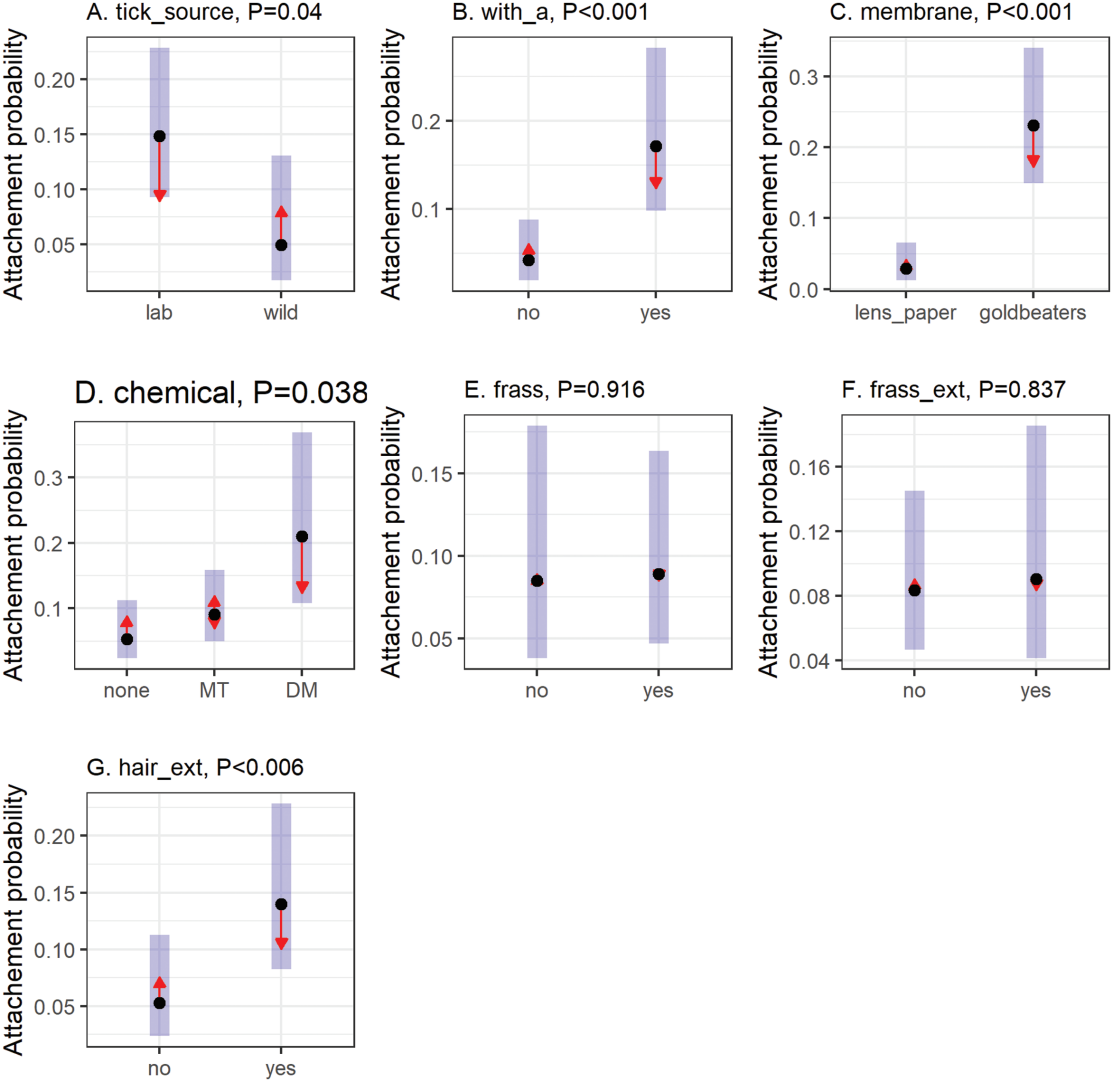
Multivariate regression analysis to determine the impact of A) different sources of *A. americanum* ticks [lab (laboratory-reared) and wild (field-collected)], B) adult *A. americanum* ticks, C) Goldbeater’s skin or lens paper membrane material, D) hair extract solutions prepared by different chemicals [DM (dichloromethane) and MT (methanol)], E) application of tick frass, F) application of tick frass extract, and G) application of hair extract. Hair type (deer or rabbit) was not included with multivariate analysis. Multivariate binomial regression fitted attachment probability values, 95% confidence intervals ( shading), and comparison arrows based on Tukey method were extracted from models using the emmeans package. Comparison arrows represent pairwise comparisons between treatments and where the arrows overlap there is no significant difference between treatments. Statistical significance for each factor is indicated.

The membrane materials and thicknesses play important roles in allowing appropriate attachment and feeding of ticks in different growth stages (Krull et al. 2017). Thus, we tested 2 different types of membranes described in prior studies and examined their thicknesses (Table 1). The thickness of lens paper membranes was 161 ± 61 µm (mean ± SD, *n* = 29) with a range of 55–300 µm measured by a digital micrometer, with one outlier (15 µm) omitted as the thickness of the lens paper was assumed to be approximately 50 µm. In contrast, Goldbeater’s membrane was much thinner, with an average thickness of 85 ± 42 µm (mean ± SD, *n* = 30) and a range of 24–178 µm. In our analysis, both membranes facilitated the attachment of adult and nymphal ticks (Fig. 3A–C). However, Goldbeater’s membrane significantly outperformed the lens paper membrane leading to higher attachment rates [odds ratio (95% CI) = 9.98(4.8–20.7), *Z* = 6.164, *P* < 0.001, Fig. 4C].

#### Host hair and hair extract.

Phagostimulants represent another important component, inducing sustained tick feeding in AFS. In our preliminary analyses, we observed that deer hair failed to enhance the attachment and feeding of *A. americanum* nymphs (Fig. 3H). On the other hand, rabbit hair exhibited variable capacities in inducing *A. americanum* feeding with additional benefits: ample supply with easy access, white color for easy detection of unfed nymphs, and guaranteed absence of pesticide treatments. Because of the small sample size (2 units with 40 ticks) tested with deer hair, we could not calculate statistical significance comparing the attachment rates for AFS performed with deer or rabbit hair.

Adding rabbit hair extract and the extraction methods improved *A. americanum* tick attachment rates. Multivariate regression analysis determined that adding rabbit hair extract provided statistically significant improvement in the attachment rates [odds ratio (95% CI) = 2.62(1.36–6.26), *Z* = 2.755, *P* = 0.0059, Figs. 3G and 4G]. For example, the combination of Goldbeater’s membrane and the addition of adult ticks and rabbit hair extract significantly improved the attachment rates for nymphal ticks [62 ± 3% with rabbit hair extract (13 units with 121 ticks) vs. 30 ± 6% without rabbit hair extract (5 units with 51 ticks), Table 1] Additionally, multivariate regression analysis identified a statistically significant improvement in units pre-treated with rabbit hair extract prepared with dichloromethane compared to those prepared with methanol [odds ratio (95% CI) = 2.68(1.04–6.86), *Z* = 2.447, *P* = 0.0382, Figs. 3D and 4D].

#### Frass and frass extract.

The addition of tick frass and frass extract did not significantly improve the experimental outcomes for nymphs, exhibiting comparable attachment rates [*frass* odds ratio (95% CI) = 1.05(0.402–2.76), *Z* = 0.105, *P* = 0.9163; *frass extract* odds ratio (95% CI) = 1.09(0.484–2.45), Z = 0.206, *P* = 0.8370, Figs. 3E, F and 4E, F]. The addition of tick frass and frass extract reduced the attachment rates in units assembled with Goldbeater’s membrane and adult ticks [46 ± 3% with frass, frass extract, and hair extract (5 units with 50 ticks) vs. 72 ± 5% with hair extract only (8 units with 73 ticks), Table 1]. Of note, one major disadvantage of adding frass or frass extract was frequent mold growth within the feeding chamber (Supplementary Table S1).

#### Addition of adult ticks to nymphal AFS units.

Incubation of adult lone star ticks with nymphal ticks in the same AFS units significantly improved the attachment and feeding rates. Despite observed variability, the addition of adult ticks enhanced the nymphal attachment rates in bivariate (Fig. 3B) and multivariate comparisons [odds ratio (95% CI) = 4.72(2.19–10.2), *Z* = 3.969, *P* = 0.0001, Fig. 4B]. For those units assembled with Goldbeater’s membrane, the addition of adult ticks improved the attachment rate [45 ± 5% with adults (18 units with 174 ticks) vs. 30 ± 5% without adults (6 units with 61 ticks), Table 1]. We also observed that nymphal ticks often fed close to adult ticks found in the peripheral edges of the feeding chamber (Fig. 2B).

#### Other modifications.

Factors other than the membrane material and feeding stimuli can play a role in the attachment of *A. americanum* ticks to the membranes and subsequent feeding. Prior studies described additional modifications, such as adding mesh or plastic crosses to the feeding chamber, to enhance the tick attachment rates (Kröber and Guerin 2007, Bullard et al. 2016, González et al. 2021). In our experiments, units assembled with fiberglass mesh did not improve the attachment and feeding rates compared to those assembled without the mesh (Fig. 2E and Supplementary Table S1). Further, the addition of mesh interfered with our attempts to inspect the status of tick feeding and removal of engorged nymphal ticks. Thus, we did not pursue this technique further.

#### Tick sources.

Laboratory-reared *A. americanum* ticks have been fed on rabbits for generations, whereas field-collected *A. americanum* ticks primarily parasitize on white-tailed deer. Therefore, we aimed to test whether AFS supports the attachment and feeding of field-collected *A. americanum*. Our results demonstrate that field-collected ticks successfully fed on the artificial membrane. However, the attachment rates for field-collected ticks remained below 30% (Table 1, Fig. 3A), which was significantly lower than the rates achieved by laboratory-reared *A. americanum* nymphs [odds ratio (95% CI) = 3.34(1.06–10.5), *Z* = 2.056, *P* = 0.0398, Fig. 4A].

### Application of the Optimized AFS for *A. americanum* Infection with *R. amblyommatis*

Recent tick surveys documented that a large proportion of *A. americanum* ticks serve as a vector and reservoir for *R. amblyommatis*. Thus, we applied the optimized AFS experimental conditions developed in this study (Goldbeater’s membrane with rabbit hair and hair extract prepared with methanol) to determine whether laboratory-reared *A. americanum* adults and nymphs (free of *R. amblyommatis*) can be stably infected with *R. amblyommatis*. The overall feeding rates for adult ticks were 14% and 0% in *R. amblyommatis*-infected and mock-infected groups, respectively (Table 2). On the other hand, the feeding rates for nymphs were much higher at 40% for *R. amblyommatis*-infected group and 56% for the mock-infected group. Among those fed, *R. amblyommatis*-specific PCR analysis determined the presence of rickettsial organisms in all adult ticks at 5- and 14-days post-infection (Table 2). Similarly, our PCR analysis confirmed the presence of *R. amblyommatis* in almost all nymphal ticks exposed to *R. amblyommatis* at both time points. The same analysis failed to detect the presence of *R. amblyommatis* in mock-infected ticks.

**Table 2.** *A. americanum* infection with *R. amblyommatis*

Groups^a^	Units^b^	Adults^c^		Nymphs^c^		Incubation days^d^	Infection (%)^e^	
		Unfed	Fed	Unfed	Fed		Adults	Nymphs
*R. amblyommatis*	3	16	2	20	10	5	100	100
Mock infection	1	6	0	0	10	5	−	0
*R. amblyommatis*	4	8	2	22	18	14	100	90
Mock infection	4	10	0	25	15	14	−	0

^a^Two experimental groups.

^b^Total number of AFS units.

^c^Total number of ticks.

^d^Incubation period following blood feeding.

^e^Percent of ticks infected with *R. amblyommatis* as determined by PCR analysis.

## Discussion

Several AFS experimental settings have been described and examined for nearly 20 different tick species (Waladde et al. 1991, Kröber and Guerin 2007, Crispell et al. 2019, Vimonish et al. 2020, 2021, González et al. 2021, Garcia Guizzo et al. 2023, Sebastian et al. 2023). However, a significant knowledge gap exists in understanding the specific experimental conditions and reagents facilitating efficient attachment and subsequent feeding for individual tick species. For instance, AFS for *I. ricinus* and *I. scapularis* have been extensively characterized and described in detail by multiple groups of investigators (Andrade et al. 2014, Oliver et al. 2016, González et al. 2021, Militzer et al. 2021, Garcia Guizzo et al. 2023). In contrast, the development of AFS for other medically relevant tick species, in particular *Amblyomma* spp. (Sebastian et al. 2023), has been delayed, partly due to the existing technical difficulties in developing AFS and working with ticks under laboratory conditions. Only 2 prior studies reported AFS applications for *A. americanum* despite the public health significance of this tick species (Bullard et al. 2016, Li et al. 2019). Both studies successfully utilized AFS for characterizing proteomic contents of cement cones and the biological functions of inward rectifier potassium channels in *A. americanum*. However, neither of these studies described the AFS methodology in detail. Therefore, this study was designed to develop and evaluate AFS for *A. americanum* and to systematically compare different materials, attractants, and other factors influencing tick attachment to artificial membranes. Using the optimized AFS, we aimed to determine whether native pathogens can be acquired by *A. americanum* using *R. amblyommatis* as a model organism.

The AFS optimization requires rigorous and iterative testing with diverse membrane materials and attachment stimuli. The most common setting employs a 6-well plate design and an artificial membrane made of lens paper impregnated with silicone rubber, creating a liquid barrier and mimicking skin texture (Kröber and Guerin 2007, González et al. 2021). An alternative material to lens paper is Goldbeater’s (or Baudruche) membrane (Krull et al. 2017, González et al. 2021). One notable difference between the 2 membranes is the membrane thickness, which should be less than the length of the tick mouthparts for proper blood feeding. As demonstrated in our study, thicker lens paper membranes are suitable for adult ticks, whereas thinner Goldbeater’s membranes support the blood feeding of immature ticks. The choice of membrane material is important not only for tick attachment but also for detachment, since engorged ticks often have to be manually removed from the membrane without causing damage to the ticks (Bullard et al. 2016). In our experience, Goldbeater’s membrane allowed the nymphs to be removed readily from the membrane. Another aspect to consider in choosing between the 2 membranes is the capacity to withstand autoclaving sterilization, which is suitable for lens paper but not for Goldbeater’s membrane. Thus, the surface of Goldbeater’s membrane often gets disinfected with 70% ethanol (Krull et al. 2017). Membrane and blood contaminations pose a significant problem in completing blood feeding using AFS. While this is less of a concern for nymphal *A. americanum* ticks reaching full engorgement within a week, adult *A. americanum* ticks require much longer periods of feeding (up to 2–3 weeks) and increased levels of protection from contaminations, warranting lens paper as a preferred membrane material (Bullard et al. 2016).

Prior studies documented the application of several tick attachment attractants and phagostimulants, such as host hair, hair extract, tick frass, frass extract, and fiberglass meshes (Kröber and Guerin 2007, Andrade et al. 2014, Bullard et al. 2016, Oliver et al. 2016, Li et al. 2019, González et al. 2021, Militzer et al. 2021). Among those, host hair has been consistently used as a necessary component of AFS. For instance, previous *A. americanum* AFS employed host hair as a sole feeding stimulant (Bullard et al. 2016, Li et al. 2019). Laboratory-raised *A. americanum* ticks are fed on rabbits (Levin and Schumacher 2016). In contrast, the white-tailed deer is the principal host for all stages of *A. americanum* in the wild (Kollars et al. 2000, Paddock and Yabsley 2007). By testing deer and rabbit hair in AFS, our results demonstrate that both hair samples induced adult *A. americanum* attachment, but only rabbit hair enhanced the attachment of nymphal ticks (Table 1). In addition, deer hair comes with several distinct disadvantages: (i) difficulties in obtaining samples (e.g., roadkill), (ii) potential contaminations with pesticides or other chemicals, and (iii) dark hair color, making it difficult to observe, locate, and remove unfed nymphs. In contrast, rabbit hair has several advantages: (i) easily obtainable from pathogen-free laboratory facilities, (ii) free of potential contaminants, (iii) white in color, and (iv) derived from a native host for laboratory-reared *A. americanum* ticks, making it suitable for AFS optimization.

Hair extract solutions are often applied to the artificial membrane with or without hair in the feeding chamber (Kröber and Guerin 2007, Andrade et al. 2014, González et al. 2021, Militzer et al. 2021). Nevertheless, some studies, including those reporting successful *A. americanum* feeding on artificial membranes, did not use any hair extracts (Bullard et al. 2016, Oliver et al. 2016, Li et al. 2019). When combined with Goldbeater’s membrane and adult ticks, but without tick frass or frass extract, hair extract significantly increased the attachment of laboratory colony *A. americanum* nymphs in our study (26 ± 4% without hair extract vs. 46 ± 3% with hair extract, Table 1).

Hair extract can be prepared with different chemicals, such as dichloromethane (Krull et al. 2017, Hart et al. 2018, Koci et al. 2018) or methanol (Dukes and Rodriguez 1976, Tajeri et al. 2016). In our experiments, both extraction methods generated good tick attachment rates. In feeding chambers prepared with Goldbeater’s membrane, adult ticks, and frass, the dichloromethane extraction method significantly improved the attachment rate (89 ± 4% dichloromethane vs. 67 ± 6% methanol). However, this modest improvement cannot be justified to offset the advantages of using methanol for preparing hair extraction solutions: (i) lower toxicity and (ii) the ability to use plastic pipettes and other plastic laboratory tools. Another extraction method using chloroform–methanol has been described in the literature and can be explored in future investigations (Militzer et al. 2021).

In our experiments, adding tick frass and tick frass extract did not improve the attachment rates for nymphal *A. americanum* (Fig. 4). In contrast, for some tick species, such as *I. scapularis*, tick frass or tick frass extract was essential for a successful feeding (Oliver et al. 2016). Unlike firm and distinct pellets of *I. scapuralis* frass, *A. americanum* produces small quantities of semi-liquid frass requiring established facilities with tick colonies to supply sufficient frass for AFS. Another significant downside of combining *A. americanum* frass extract with host hair is increased levels of fungal contaminations on the artificial membrane, as observed in our experiments and prior studies (Militzer et al. 2021). In future experiments, additional steps, including filter sterilization of the extract, storage at −20°C, and application of antifungal agents, can be considered to prevent excessive mold growth.

In other experiments, supplementary attachment enhancements (for instance, mesh and other armature) have been utilized for AFS (Kröber and Guerin 2007, González et al. 2021). However, there exist conflicting experimental outcomes in the literature concerning supplementary attachments. For instance, Bullard et al. engineered AFS supplemented with glued fiberglass mesh and successfully used it for *A. americanum* feeding (Bullard et al. 2016). In contrast, Li et al. (2019) reported that AFS for *A. americanum* did not require additional mechanical stimuli. Similarly, we did not observe noticeable improvements after applying fiberglass mesh in early trials (Supplementary Table S1). In fact, the mesh made it more challenging to locate and detach engorged nymphs and was thus excluded from further experiments. However, it remains to be explored whether mesh or other armature is beneficial for improving adult *A. americanum* attachment to artificial membranes, as has been observed for other tick species (Militzer et al. 2021).

Blood contamination is rarely reported but is a common problem affecting the outcomes of AFS tick feeding. If the feeding units placed on top of a 6-well plate fail to seal the opening completely, frequent bacterial and fungal contaminations occur as the entire unit is constantly exposed to high humidity and warm temperature. In our experience, placing AFS within a sealed plastic box did not prevent blood contamination. Further, washing the membrane and replacing the 6-well chamber did not help remove contaminating agents. However, adding antibiotics and antifungal agents reduced the occurrence of blood contamination (data not shown). Of note, some of our feeding experiments were conducted without antibiotics and antifungal agents, demonstrating the feasibility of completing nymphal tick feeding. However, it will pose a serious impediment to adult AFS development (requiring a longer feeding period) and pathogen transmission studies. Thus, fundamental mechanical improvements to the AFS design are essential to mitigate this issue.

The main focus of this study was to develop AFS for *A. americanum* nymphs. The optimized AFS system for nymphs will enable investigators to study the underlying mechanisms involved in horizontal and transstadial transmissions for diverse tick-borne pathogens. Despite these advantages, published information on AFS designed specifically for *A. americanum* nymphs is scarce, with Bullard et al. (2016) the only study reporting nymphal feeding. In addition to the attachment stimuli discussed above, the feeding efficacy of *A. americanum* nymphs was increased when several adult lone star ticks were added to the same feeding chamber (Fig. 4). Lone star nymphs congregated near attached adult ticks, possibly due to the pheromones released by adults (Yoder et al. 2008). Thus, our studies identified 3 critical components of AFS required for nymphal *A. americanum* tick attachments: (i) Goldbeater’s membrane, (ii) rabbit hair and hair extract prepared with methanol, and (iii) addition of adult lone star ticks to nymphal feeding chambers. With this optimized setting, we observed consistent nymphal tick attachment (46 ± 3%) and feeding (100%, i.e., one or more attached ticks in each feeding chamber), achieving > 90% infection with *R. amblyommatis*, a spotted fever group rickettsial organism frequently found in field-collected *A. americanum*.

Our work adds to the field by demonstrating the feasibility and utility of AFS for studying tick-borne pathogen transmission, host–tick–pathogen interactions, and tick control methods (Kröber and Guerin 2007, Migné et al. 2022). Building on our work, future studies may consider testing additional combinations of physical and chemical components and optimizing AFS for other medically important tick species. For instance, a newly engineered instrument can test whether release of carbon dioxide into the feeding chamber and/or differential temperature gradient across the feeding chamber improve the attachment and feeding rates even further. Additionally, optimal membrane thickness and timing of feeding experiments (for instance, within or outside of the natural active season) remain to be investigated. These improvements may assist reducing variabilities observed in our experiments and increase the power of individual experiments without the need of large number of ticks and labor-intensive procedures. With the optimization of AFS, additional studies are necessary to determine the differences and similarities in transovarial and transstadial transmission of pathogens, such as *R. amblyommatis*, in *A. americanum* fed on artificial membranes or animal hosts. These studies will provide additional insights into the understanding of host-tick-pathogen interactions.

## Supplementary Material

tjad158_suppl_Supplementary_Tables_1
